# Response to COVID-19 in South Korea and implications for lifting stringent interventions

**DOI:** 10.1186/s12916-020-01791-8

**Published:** 2020-10-09

**Authors:** Amy Dighe, Lorenzo Cattarino, Gina Cuomo-Dannenburg, Janetta Skarp, Natsuko Imai, Sangeeta Bhatia, Katy A. M. Gaythorpe, Kylie E. C. Ainslie, Marc Baguelin, Samir Bhatt, Adhiratha Boonyasiri, Nicholas F. Brazeau, Laura V. Cooper, Helen Coupland, Zulma Cucunuba, Ilaria Dorigatti, Oliver D. Eales, Sabine L. van Elsland, Richard G. FitzJohn, William D. Green, David J. Haw, Wes Hinsley, Edward Knock, Daniel J. Laydon, Thomas Mellan, Swapnil Mishra, Gemma Nedjati-Gilani, Pierre Nouvellet, Margarita Pons-Salort, Hayley A. Thompson, H. Juliette T. Unwin, Robert Verity, Michaela A. C. Vollmer, Caroline E. Walters, Oliver J. Watson, Charles Whittaker, Lilith K. Whittles, Azra C. Ghani, Christl A. Donnelly, Neil M. Ferguson, Steven Riley

**Affiliations:** 1 MRC Centre for Global Infectious Disease Analysis, Abdul Latif Jameel Institute for Disease and Emergency Analytics (J-IDEA), Imperial College London, London, UK; 2 NIHR Health Protection Research Unit in Healthcare Associated Infections and Antimicrobial Resistance, Imperial College London, London, UK; 3 School of Life Sciences, University of Sussex, Brighton, UK; 4 Department of Laboratory Medicine and Pathology, Brown University, Providence, RI USA; 5 Department of Statistics, University of Oxford, Oxford, UK

**Keywords:** COVID-19, South Korea, Public health interventions, Reproduction number, Contact tracing

## Abstract

**Background:**

After experiencing a sharp growth in COVID-19 cases early in the pandemic, South Korea rapidly controlled transmission while implementing less stringent national social distancing measures than countries in Europe and the USA. This has led to substantial interest in their “test, trace, isolate” strategy. However, it is important to understand the epidemiological peculiarities of South Korea’s outbreak and characterise their response before attempting to emulate these measures elsewhere.

**Methods:**

We systematically extracted numbers of suspected cases tested, PCR-confirmed cases, deaths, isolated confirmed cases, and numbers of confirmed cases with an identified epidemiological link from publicly available data. We estimated the time-varying reproduction number, *R*_*t*_, using an established Bayesian framework, and reviewed the package of interventions implemented by South Korea using our extracted data, plus published literature and government sources.

**Results:**

We estimated that after the initial rapid growth in cases, *R*_*t*_ dropped below one in early April before increasing to a maximum of 1.94 (95%CrI, 1.64–2.27) in May following outbreaks in Seoul Metropolitan Region. By mid-June, *R*_*t*_ was back below one where it remained until the end of our study (July 13th). Despite less stringent “lockdown” measures, strong social distancing measures were implemented in high-incidence areas and studies measured a considerable national decrease in movement in late February. Testing the capacity was swiftly increased, and protocols were in place to isolate suspected and confirmed cases quickly; however, we could not estimate the delay to isolation using our data. Accounting for just 10% of cases, individual case-based contact tracing picked up a relatively minor proportion of total cases, with cluster investigations accounting for 66%.

**Conclusions:**

Whilst early adoption of testing and contact tracing is likely to be important for South Korea’s successful outbreak control, other factors including regional implementation of strong social distancing measures likely also contributed. The high volume of testing and the low number of deaths suggest that South Korea experienced a small epidemic relative to other countries. Caution is needed in attempting to replicate the South Korean response in populations with larger more geographically widespread epidemics where finding, testing, and isolating cases that are linked to clusters may be more difficult.

## Background

**Fig. 1:**
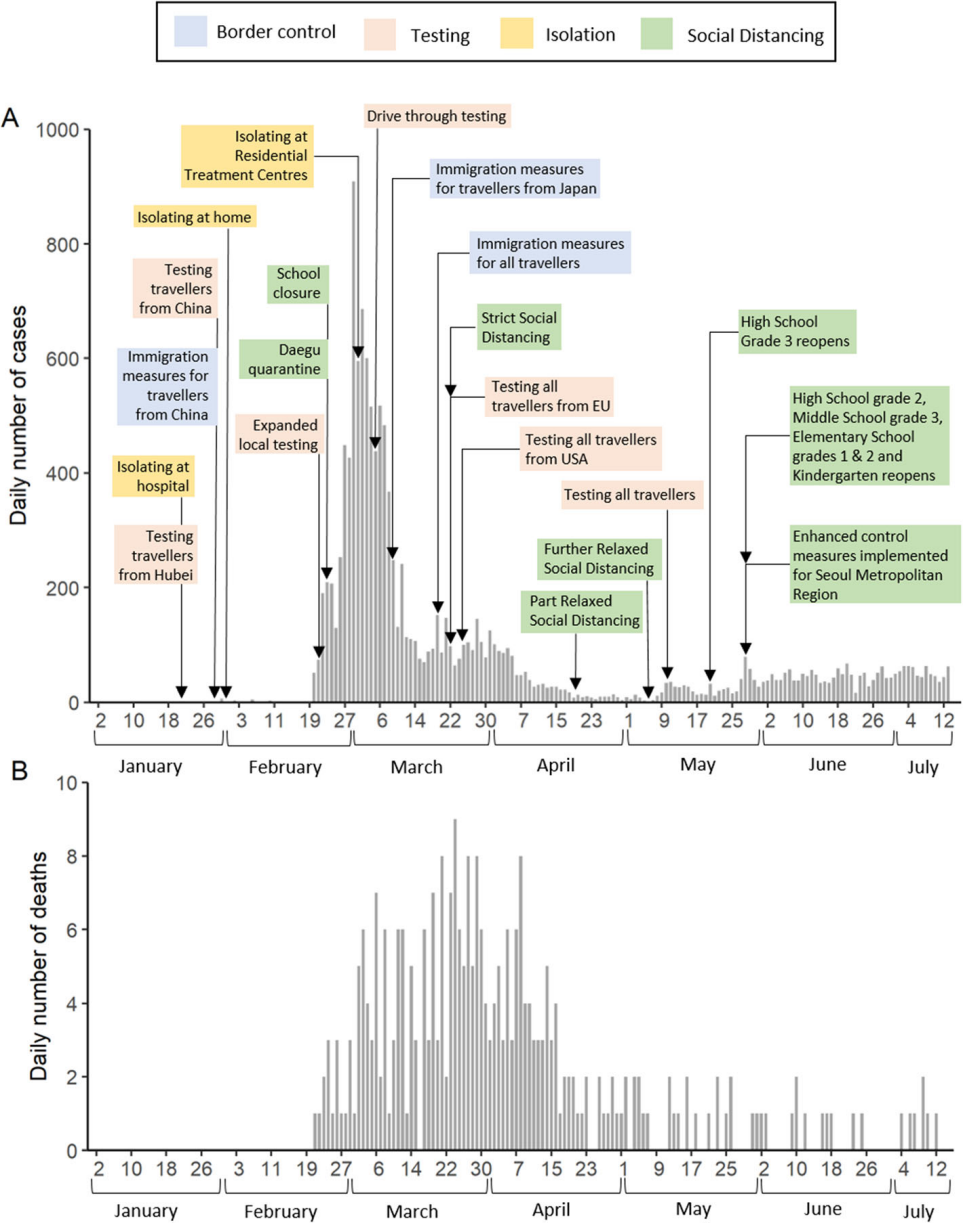
Incidence of confirmed cases by date of the report from daily KCDC press releases with key intervention timings (**a**) and deaths (**b**). Interventions are separated into border control, testing, isolation and social distancing. Sources of interventions: KCDC press releases, Ministry of Health and Welfare, and Ministry of Education

Coronavirus disease 2019 (COVID-19) has become a global pandemic with 18,519,579 cases and 700,539 deaths reported as of August 5, 2020 [1]. One of the first countries to be affected by COVID-19 was South Korea, where the first confirmed case was reported on January 20, 2020, detected at Incheon International Airport [2]. Prior to February 14th, the majority of the 28 reported cases were imported or were household contacts of imported cases [3]. Following identification of the cluster of local transmission amongst members of the Shincheonji religious group in Daegu linked to the 31st reported case in the country on February 20th, testing was expanded to suspected cases based on the physicians’ judgement, including individuals with no travel history [4]. Case incidence peaked on February 29th, shortly after Daegu and Cheongdo were declared “special management regions” and citizens were asked to refrain from leaving their homes for at least 2 weeks [5, 6] (Fig. 1a and Additional file 1: Table S1) [4, 7–35] and has since generally been declining. The decline started approximately 3 weeks before stricter social distancing measures (including stay-at-home recommendations and restriction of non-essential retail) were implemented nationally [7]. As of July 13th, South Korea has reported a total of 13,479 cases with 62 new cases on the last day. The pattern of COVID-19 deaths in South Korea has not followed the same clear epidemic pattern as cases (Fig. 1b), with a total of only 289 deaths up to and including July 13th, distributed throughout the period since February 20th with a peak, by date of the report, of only 9 deaths per day.

Despite a rapid growth of cases early in the global timescale, South Korea has brought the transmission of the causative agent of COVID-19 (severe acute respiratory syndrome coronavirus 2 (SARS-CoV-2)) under control with less stringent national social distancing policies relative to countries such as Italy, France, and the UK [36]. This has led to substantial interest in their “test, trace, isolate” strategy, as other countries begin to relax their “lockdowns” and transition to more sustainable packages of interventions. However, it is important to understand the epidemiological peculiarities of the South Korea outbreak and characterise their effective response package before trying to apply these measures to other settings.

We conducted a quantitative review of South Korea’s interventions in response to the COVID-19 epidemic to draw insights into the different components of the response. We characterised temporal trends in (i) transmission, by estimating the time-varying reproduction number R; (ii) testing capacity; (iii) case origins and how they were identified; and (iv) case isolation. We discuss possible implications for other countries’ strategies for lifting stringent social distancing interventions.

## Methods

### Data

We systematically reviewed each daily update on COVID-19 in South Korea published by the Korea Centers for Disease Control and Prevention (KCDC) for the period from January 20 to July 13, 2020 [37]. Using a mixture of manual extraction and automated data scraping scripts written in R software, we extracted the number of suspected cases being tested, PCR-confirmed cases (polymerase chain reaction), deaths and recoveries, and confirmed cases in isolation for each day, forming a time series. Where available, we also manually extracted the number of imported cases per day (from March 30th onwards) and the numbers of confirmed cases with an identified epidemiological link and/or origin to assess the efficacy of contact tracing and identification of transmission clusters (from March 25th onwards). When unable to calculate statistics of epidemiological importance such as delays to case-based interventions from our extracted data, we reviewed the published literature to attempt a holistic review of the interventions implemented by South Korea. We also utilised announcements by the KCDC, the Ministry of Health and Welfare (MOHW), and the Ministry of Education to identify policy changes to complement our quantitative time series data.

### Time-varying reproduction number, *R*_*t*_

We used an approach similar to those previously described [38, 39] to quantify transmissibility over a 7-day sliding window, from the case incidence time series, assuming a certain distribution for the serial interval (the time between symptom onset in a case and symptom onset in their infector). We assumed that transmissibility was constant over that 7-day window and measured it through the reproduction number, *R*_*t*_, defined as the average number of secondary cases infected by an infected individual. Our method accounts for imported cases, which would otherwise tend to inflate *R*_*t*_, as previously described [40], and assumes that the daily incidence of locally acquired cases can be approximated by a Poisson process using the so-called renewal equation:

$$ {I}_t^{\mathrm{local}}\sim \mathrm{Poisson}\left({R}_t{\sum}_{s=1}^t{I}_{t-s}{w}_s\right) $$

where ${I}_t^{\mathrm{local}}$ is the “true” incidence of locally acquired cases on day *t*, *R*_*t*_ is the reproduction number on day *t*, *I*_*t* − *s*_ is the total (local and imported) number of incident cases arising *s* days before day *t*, and *w* is the probability mass function of the generation time. Since the date of infection is rarely known, when estimating *R*_*t*_ case, incidence based on the date of symptom onset is often used in place of true incidence and the serial interval in place of the generation time. As onset dates were not available for all cases in our dataset, *R*_*t*_ was estimated based on cases by date of the report using data from KCDC using the R software packages *EpiEstim* and *orderly.* All analyses were undertaken with R software (version 3.6.3) [38, 41–43]. We assumed a gamma-distributed serial interval with mean 6.48 days and standard deviation 3.83 days [44] and uninformative prior distributions on *R*_*t*_ (mean 1 and standard deviation 10). To assess the sensitivity of the *R*_*t*_ estimates to the assumed prior distribution of *R*_*t*_, we ran our analysis with a range of prior distributions (Additional file 1: Table S3) and found that our conclusions were consistent.

*EpiEstim* is robust to under-reporting but assumes that this reporting rate remains constant. This was not the case in South Korea due to the changes in the case definition and testing criteria, most notably the scale-up in testing to include local transmission from February 20th (Fig. 3). This causes bias in the *R*_*t*_ estimate during the period in which testing processes changed. We truncated the data to conservatively exclude this period of bias following the scale-up in testing and present *R*_*t*_ estimates from March 30th onwards to align with the period for which imported case numbers are available.

### Positive cases per test

To calculate the rolling 7-day average of cases per test, we first calculated the right-aligned 7-day rolling average of cases and tests separately using the *roll_mean* function from the R software package *RcppRoll* [45]. We then divided the average number of cases by the average number being tested for each day to obtain an estimate for the average number of cases per test. We chose to calculate the rolling average for the number of people being tested, as testing varied considerably over consecutive days, with no tests conducted some days. We also calculated the rolling average for 3-, 4-, 5-, and 6-day windows, but window size did not impact the general trend.

### Epidemiological links and origins of confirmed cases over time

KCDC press releases break down cumulative numbers of cases into imported cases, linked to an imported case, part of Shincheonji cluster, part of another cluster, a contact of a confirmed case, or under investigation. We used this to look at the change in the cumulative proportion of cases attributed to different categories over time. As epidemiological investigations develop, there is a bidirectional movement of cases between categories, so it was not possible to create a time series of case incidence by type. Instead, we extracted data from the pie chart showing the types of cases over the 2 weeks prior to the date of each press release. This provided us with a 14-day rolling average breakdown of incident cases by type.

## Results

### Transmission

**Fig. 2:**
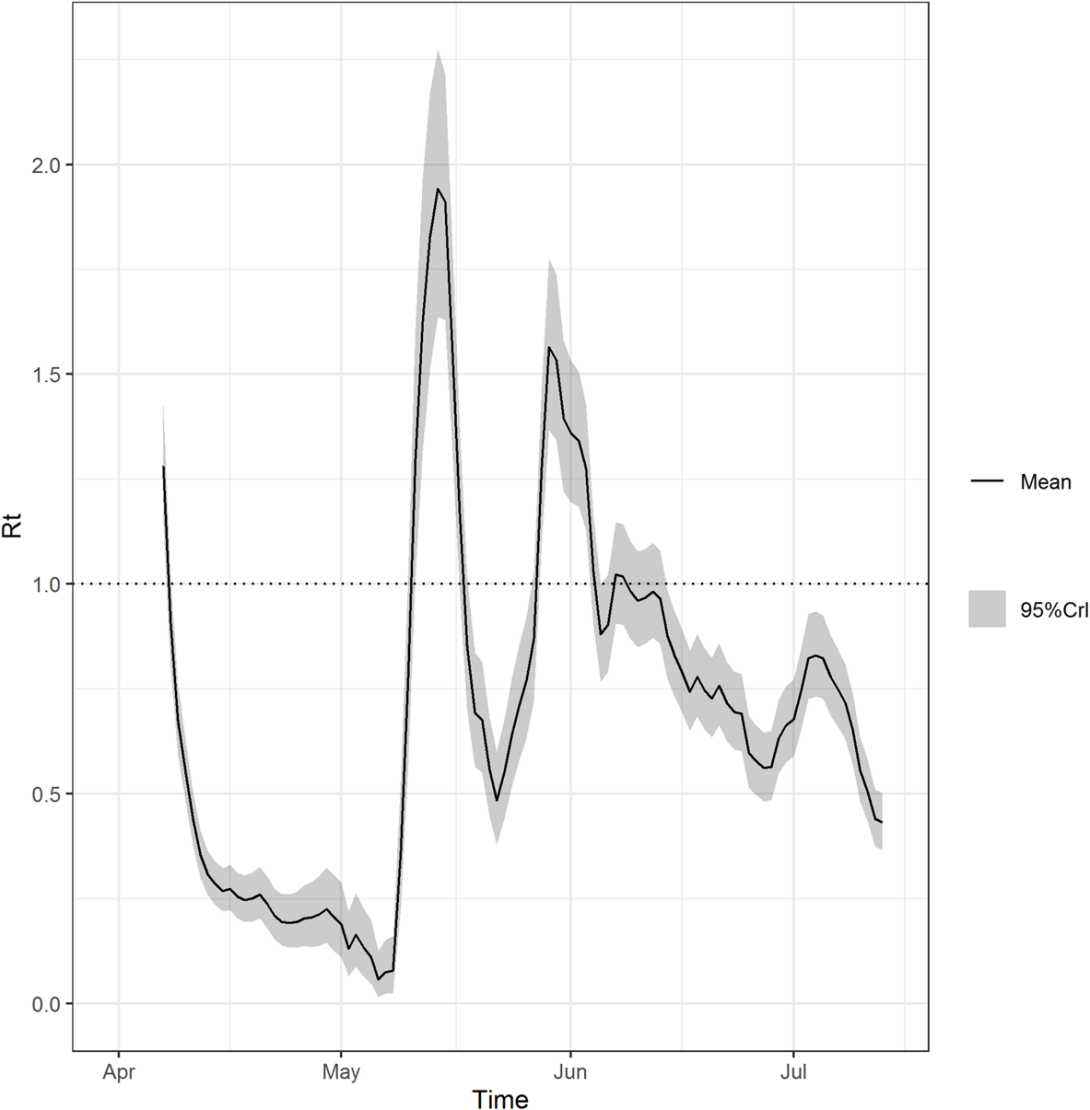
Estimates of time-varying effective reproductive number *R*_*t*_ based on confirmed cases by date of the report and accounting for imported cases. *R*_*t*_ is estimated using a sliding window of 7 days and an uninformative prior distribution with mean 1 and standard deviation 10. The black line represents the posterior mean value of *R*_*t*_, and the shaded region shows the 95% credible interval (CrI). The dotted horizontal line shows *R*_*t*_ = 1

Our estimates of SARS-CoV-2 transmissibility in South Korea vary substantially over time (Fig. 2). We estimated the time-varying reproduction number *R*_*t*_ using previously published methods [38] for cases between March 30th and July 13th, since the relative contributions to *R*_*t*_ from increased testing and increased transmission cannot be disentangled early on in the epidemic and imported case numbers were not initially available. Using a 7-day sliding window and confirmed cases by date of report, we estimated that *R*_*t*_ initially dropped below 1 in early April before increasing above one twice during May, first up to a maximum of 1.94 (95% CrI, 1.64–2.27) and then again in late May up to 1.56 (95% CrI, 1.37–1.79) (Fig. 2). These increases in estimated *R*_*t*_ coincide with the growth of highly localised transmission clusters in the Seoul Metropolitan Region (Seoul, Incheon city, and Gyeonggi province). In early June, *R*_*t*_ dropped below 1 again where it remained until the end of our study on July 13th.

### Interventions implemented by South Korea

#### Social distancing

Unlike other countries affected by COVID-19, South Korea has not implemented a national “lockdown”. However, similar measures have been applied in a short-term localised manner in high-incidence regions such as Daegu where residents were asked to refrain from leaving their homes for at least 2 weeks after the large cluster of cases associated with the Shincheonji religious group was reported [6] (Fig. 1a). KCDC reported that movement decreased nationally by 38% during February 24th to March 1st as compared to January 9th to 22nd before the first case had been detected in South Korea [46, 47]. This decrease in movement is corroborated by Park et al. who found that the daily traffic volume decreased by 80% and 50% in Daegu and Seoul, respectively, following the identification of the Shincheonji cluster [47]. Schools were closed on February 23rd nationally, and the country transitioned to remote learning until schools reopened gradually between May 20th and June 3rd [12, 14, 31, 48]. On March 22nd, in response to the ongoing emergence of local small clusters, imported cases, and a reported increase in population movement up to 28% below baseline [46], the Korean government implemented a stronger social distancing campaign nationally (Fig. 1a). People were asked to leave their houses only for daily necessities, healthcare, and commuting to work, and many community spaces were closed with the potential for non-compliant facilities to be handed administrative orders [49, 50]. On April 19th, low-contact outdoor facilities and churches could reopen. On May 6th, South Korea transitioned again with the intention of establishing long-term routine distancing [34, 51]. The KCDC criteria for sustaining the routine distancing policy include low daily case numbers, low incidence of detecting apparently sporadic cases, and detection of > 80% of new cases in individuals who are already in self-quarantine. These conditions were being met until the increase in cases in the Seoul Metropolitan Region led to the daily threshold being exceeded on May 28th [52]. In response to increased local transmission, enhanced epidemic control measures have been introduced in the region until daily case numbers are back below 10 [13, 53]. High-risk facilities including nightlife venues, karaoke rooms, and PC cafes are subject to gathering bans or limited operation with potential legal action against non-compliance. Residents are requested to not attend social gatherings or venues frequented by lots of people. Following further transmission in churches, additional infection control guidelines became mandatory on July 10th including instruction not to hold face-to-face meetings outside of regular services, to check symptoms upon entry, and not to partake in singing or food sharing during services [22].

#### Testing

**Fig. 3:**
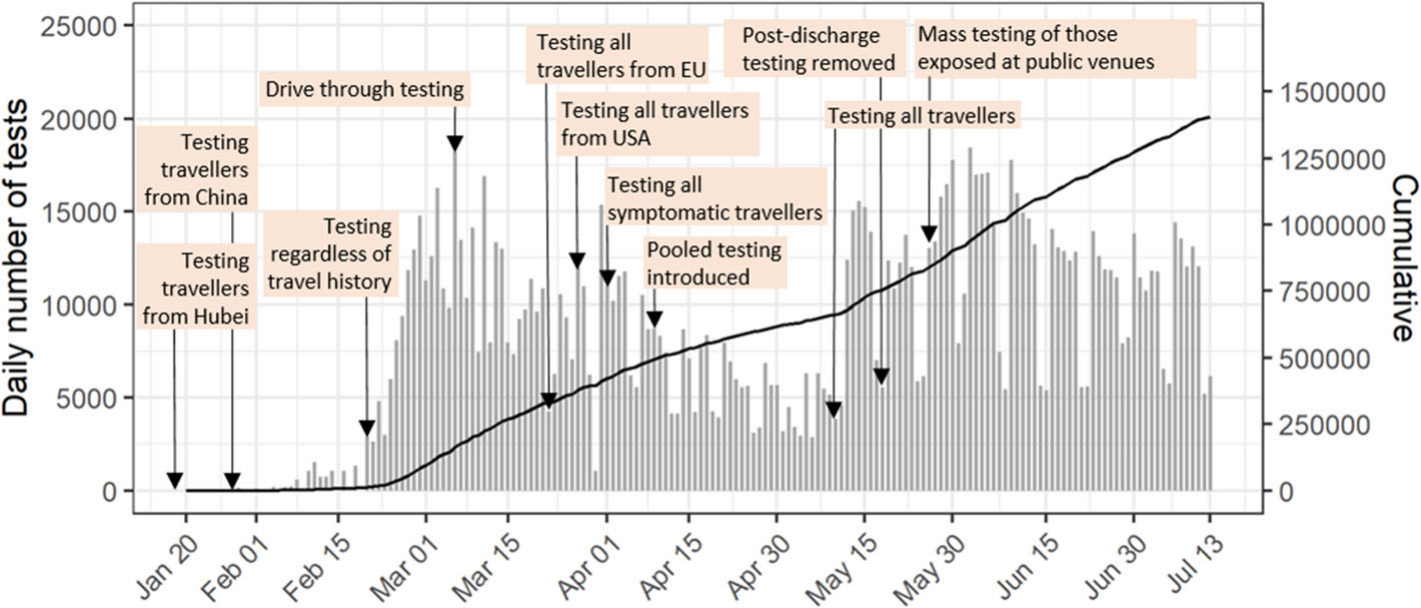
New and cumulative numbers of people being tested in South Korea from the beginning of the COVID-19 outbreak on January 20 until July 13, 2020. Grey bars represent the daily number of tests by date of the report, and black line shows the cumulative number of tests conducted over time. Key changes in testing are also presented. Source: KCDC press releases

**Fig. 4:**
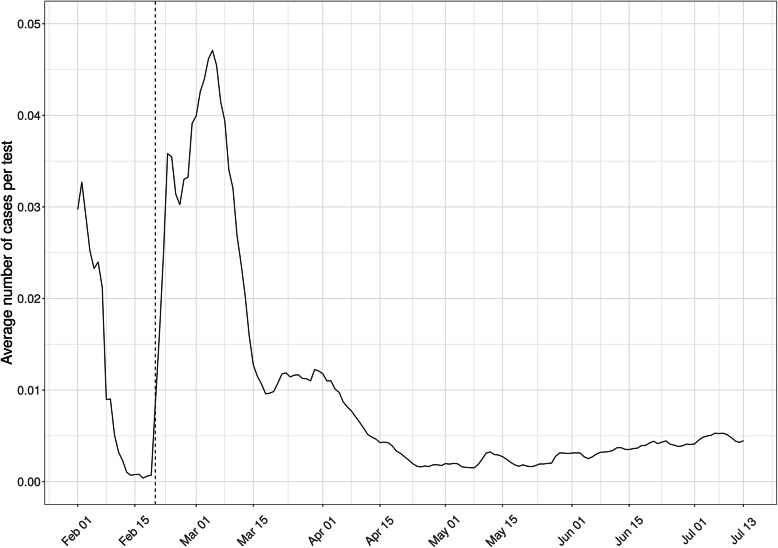
Rolling 7-day average of the confirmed cases per number of people tested until July 13, 2020. The dashed vertical line indicates the date on which South Korea changed its testing protocol from testing suspected cases with contact with a case or travel history to a country with infections to testing all suspected cases (February 20). Source: KCDC press releases

South Korea has a population of 51 million [54]. By licencing private companies and clinics to conduct tests early in the epidemic, testing capacity was rapidly expanded from 3000/day on February 7th to 15,000–20,000/day with a turnaround time of 6–24 h by the end of March [7]. As of March 25th, there were 118 institutions capable of conducting tests for COVID-19 including KCDC, 4 National Quarantine Stations, 18 Research Institutes of Public Health and Environment (RIPHEs), and 95 private medical laboratories and hospitals [4]. Testing protocol in South Korea changed multiple times throughout the outbreak (Fig. 3 and Additional file 1: Table S1, Table S2) [35, 55–58]. Initially, only symptomatic individuals with a history of travelling in areas with known infections, or contact with confirmed cases were tested [59]. However, the policy changed on February 20th to include testing of symptomatic individuals based on the physicians’ judgement regardless of travel history and to test close contacts of confirmed cases regardless of symptoms if deemed necessary [60]. This coincided with the Shincheonji religious group cluster and explosive growth in cases. The average number of positive cases per test increased during this period (Fig. 4). Mass testing has also been used in high-risk facilities such as hospitals and care homes in high incidence areas since March 18th. In Daegu, a total of 33,610 high-risk individuals have been tested with 322 testing positive as of April 4th [51]. As case numbers decreased, the average number of cases per test decreased again until the mid-May increase in cases, which resulted in another increase in the average number of cases per test. We are unable to infer the delay from symptom onset to testing from our data set collated from the KCDC daily press releases. A study of the first 28 patients (identified from January 20th to February 10th) estimated the mean delay from symptom onset to diagnosis to be 5.2 days (range 0–16 days) [61]. We did not find data on delays to testing later in the outbreak.

#### Identification of clusters and contact tracing

**Fig. 5:**
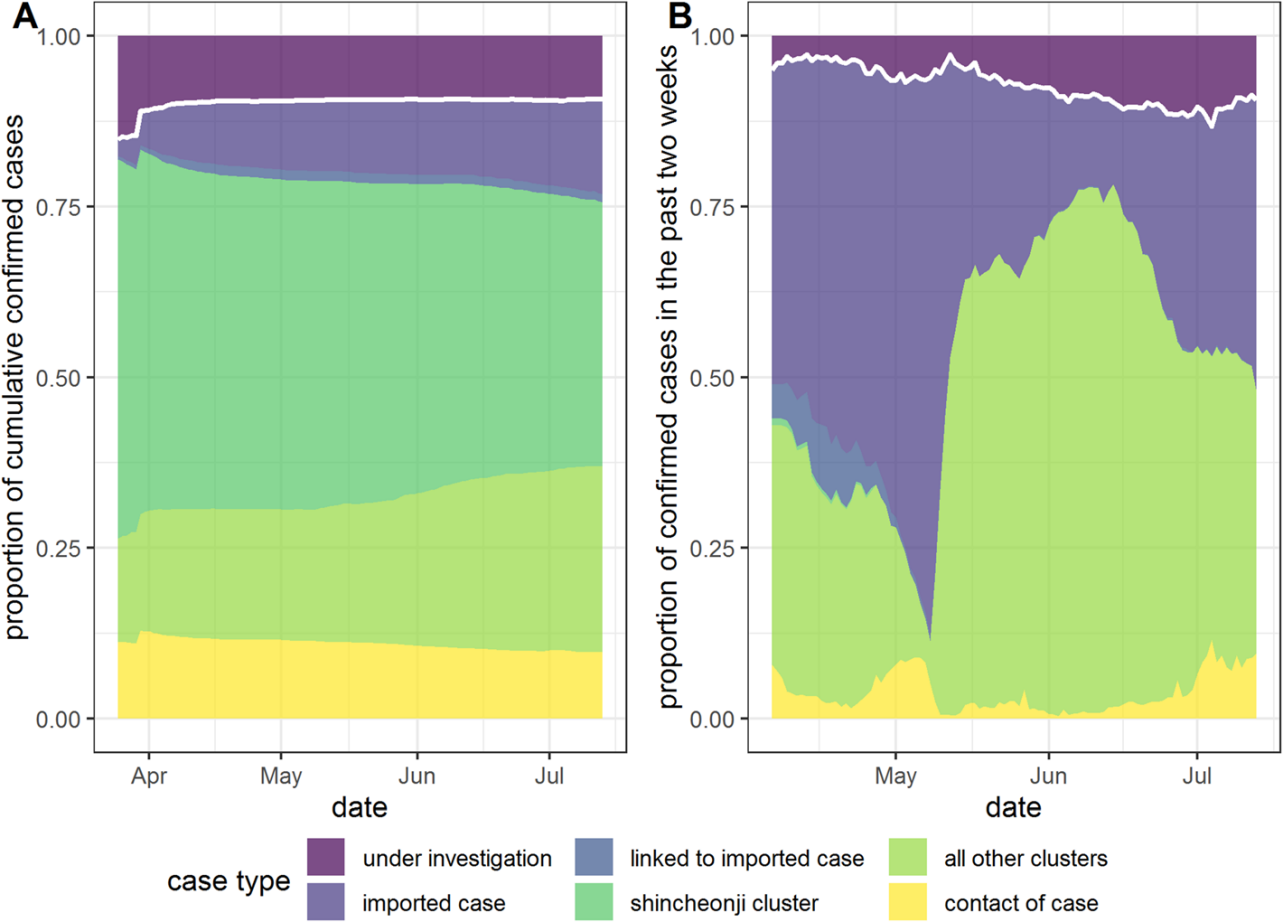
**a** Cumulative proportion of confirmed cases by epidemiological link and/or origin from March 25 to July 13. The proportion of cumulative confirmed cases that are linked to existing cases or imported (white line) as opposed to “under investigation” (which includes apparent sporadic cases). Linked cases are broken down into whether they are connected to an imported case, part of the Shincheonji cluster, a smaller cluster or a non-cluster contact of a confirmed case. **b** Non-cumulative proportion of new cases reported during the 2 weeks prior to the date on the *x*-axis, by type of case. Source: KCDC press releases

A high proportion of cases have been epidemiologically linked throughout the outbreak. Case-based contact tracing in South Korea can be separated into (i) cluster investigation utilising targeted mass testing of hospitals and communities and (ii) identification and follow-up of individual cases and their contacts (see Additional file 1: Section 2. Contact tracing for more detailed protocol) [30, 62–68]. Clusters now account for 66% of South Korea’s cumulative cases nationally, while individual case-based contacts account for only 10% (Fig. 5a). The large cluster associated with the Shincheonji religious group accounts for 39% of cases nationally. Although focused in Daegu, the cluster included group members in most regions of the country and made considerable contributions to overall case numbers in Gyeongbuk, Gyeongnam, Gangwon, Ulsan, and Gwangju (see Figure S1 for the regional breakdown of case type by epidemiological link or origin). Later in the epidemic, after the March 25th, the Shincheonji cluster contributed very few new cases. Other smaller clusters make up the further 27% of cases (ranging in size from 2 to 196 people). Prior to May 8th, these clusters were mainly in workplaces, hospitals, and churches, with most clusters of > 100 cases occurring in hospitals, where mass testing protocols were in place [69]. The relative contribution of local transmission to new cases steadily decreased until 82% of new cases in the fortnight prior to May 8th were imported; however, the Itaewon nightclub cluster [35] brought the relative contribution of local clusters back up to 40% of new cases reported in the fortnight ending on of May 11th (Fig. 5b). Since then, the majority of reported cases have been linked to clusters in the Seoul Metropolitan Region, largely associated with entertainment venues, religious events, after school private academies, and a string of door-to-door promotional events [13, 53]. The contribution of local transmission clusters to overall fortnightly case incidence reached a peak of 75% in June before beginning to decline back down to around 50% at the end of the study period (Fig. 5b).

#### Isolation

**Fig. 6:**
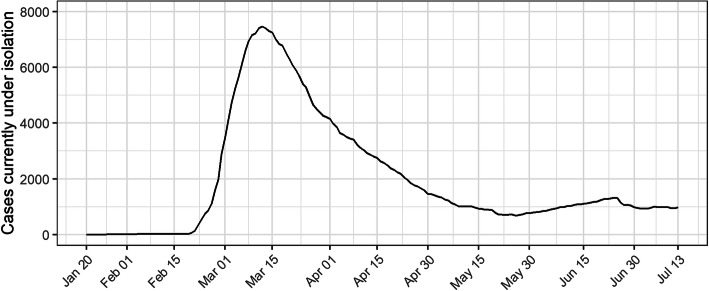
Isolation dynamics in South Korea. The number of confirmed cases currently in isolation per day. Source: KCDC press releases

Isolation of cases and self-quarantine of contacts has been a consistent feature of the South Korean response. As of July 13, there were 986 confirmed cases currently isolated (Fig. 6). Although we were unable to identify accessible data sources that reported the number of contacts isolated over the course of the outbreak, media outlets reported that approximately 30,000 people were in self-quarantine on March 6th [70], 27,000 on April 2nd [71], and 46,000 on April 7th [72]. In addition, a study of contact tracing in South Korea between January 20th and March 27th found that for the 5706 index cases with at least one contact, 59,036 contacts were identified (who according to KCDC policy would have been required to self-quarantine) [73]. Based on the severity and risk factors, confirmed cases were either isolated in a hospital, at home, or in a residential treatment centre (RTC). Cases were isolated in a hospital in their own rooms or grouped with patients with the same clinical characteristics when ventilation and structural barriers between patients were available. Cases were isolated in RTCs when symptoms were too severe for home isolation, but did not require hospitalisation, when home isolation was not possible because no individual room was available or because they live with a high-risk person [66]. Medical staff in RTCs and public health managers (for home isolation) monitored and recorded the patient’s symptoms twice per day, transferring them to medical facilities if necessary. Close contacts of confirmed cases were asked to self-quarantine for 14 days and monitored daily.

We were unable to estimate the delay to isolation of a confirmed case from the KCDC press release data. Another study, which fitted gamma distributions to case data of 211 confirmed cases, estimated the mean delay from symptom onset to isolation of cases as 4.3 days, before the red alert warning on February 23rd and 3.3 days after [74]. The same study estimated the mean delay from exposure to isolation as 7.2 days before the red alert and 6.5 days after.

Confirmed cases were required to test negative twice in 24 h before release from isolation [66]. Close contacts with no symptoms in the 14 days following contact were released from home quarantine. If close contacts were health care workers or caregivers, they were released pending a negative at the end of the quarantine period.

## Discussion

This analysis of South Korea’s response to COVID-19 highlights how, despite a sharp growth in cases early in the pandemic, transmission was rapidly controlled, resulting in a relatively small epidemic made up of large linked clusters. The Shincheonji religious group cluster accounted for 39% of all cases. We estimate *R*_*t*_ to have been brought below one in April, and although estimates rose above one during May and early June, this reflected transmissibility within highly localised clusters rather than widespread national transmission. As of July 13th, 13,479 cases and 289 deaths have been reported since the first case on January 20th [2]. The rapid expansion of test capacity, early localised strengthening of social distancing measures in Daegu, voluntary reduction in movement prior to the mandated enhanced national social distancing campaign, and continued case-based contact tracing across the large clusters in Seoul Metropolitan Region have all likely contributed to help contain South Korea’s epidemic.

South Korea has not implemented a national lockdown. Instead, the comprehensive approach ranges from case-based testing and isolation, investigation of large clusters of confirmed cases, and short, targeted strong social distancing measures in the most heavily affected regions. Early identification and management of cases, clusters, and contacts have been a key strategy of the South Korea response. Combined with the expanded testing capacity, the use of mass testing in high-risk facilities and clusters has allowed the majority of new cases to be traced back to existing clusters (Fig. 5), such as the Shincheonji religious group, a call centre in Seoul, healthcare institutions in Daegu, and the Itaewon nightclubs.

There are several limitations to this analysis. South Korea’s response was characterised by multiple measures including rapid large-scale testing and social distancing measures, and we did not find enough evidence to attribute the success in controlling the COVID-19 outbreak to one particular aspect of their response. Although it is possible to attribute changes in transmission to specific policies for a single population, it is often difficult to identify the effect of a single intervention because many are in force at the same time. Therefore, we chose not to attempt such inference here, leaving multi-population estimates of intervention efficacy to future work, similar to that of Flaxman et al. [75]. When estimating *R*_*t*_, we assumed constant reporting, which does not hold for the South Korea dataset. The testing protocol changed considerably on February 20th, and a number of imported cases were only available from March 30th onwards meaning we could only estimate *R*_*t*_ reliably between March 30th and July 13th. During this period, we assumed testing efforts to be constant; however, there were smaller protocol changes (Fig. 3 and Additional file 1, Table S1). These changes largely pertained to the expansion of testing of travellers, except for May 25th when South Korea began mass testing of individuals who visited public venues visited by confirmed COVID-19 cases. Mass testing had been part of cluster investigations previously, but this change may have impacted our estimates of *R*_*t*_. In the absence of symptom onset data, we used the date of the report as a proxy for incidence, meaning our estimates of *R*_*t*_ are subject to a lag from incubation period and delay from symptom onset to report. Estimated dates of symptom onset would improve the accuracy of *R*_*t*_ estimates for South Korea.

A dynamic transmission model could help disentangle the relative contribution of different measures and account for changes in reporting. Through a non-systematic review of the published literature and a systematic review of governmental sources (KCDC), we were able to identify important data gaps. The national-level dataset we collated did not contain information on how many contacts each individual case had (and the proportion who tested positive), although data on the total number of contacts per confirmed case were available for some municipalities [76, 77]. Furthermore, we could not estimate a delay from confirmation of test result to the isolation of cases or their contacts from our dataset. There is a need for reliable up-to-date information on the number of contacts isolated at any one time. Finally, it was unclear whether the number of suspected cases being tested each day includes individuals tested more than once, and testing of asymptomatic close contacts who would not fit within the formal definition of a suspected case.

Like other countries looking at exiting their lockdowns, the long-term strategy in South Korea involves a mix of social distancing policies, behavioural interventions, and testing. South Korea moved to a phase of partially relaxed social distancing on May 6th [34]. This included allowing activities in low-risk venues (e.g. recreation parks, forests, closed-door sporting events) while maintaining partial restrictions on the use of high-risk facilities (e.g. religious facilities, fitness venues) and non-essential public gatherings which are all subject to strict infection prevention and control guidelines [33]. Additionally, schools reopened in late May and a growing number of companies have eased their “smart-working” policies [12, 14, 48]. Consultations between government, industry, and community-based bodies have been conducted to increase preparedness, reduce conflicts, and maximise uptake of long-term “every day” social distancing [78].

It is important to emphasise some key aspects particular to the outbreak in South Korea, which are relevant when attempting to apply lessons to other settings. The number of SARS-CoV-2 infections in South Korea has been far smaller than the USA or most European countries. Over two thirds of cases were linked to a few major clusters, which were promptly identified and tested en masse. South Korea has implemented effective strategies for detecting and quarantining imported cases, reducing the risk of secondary transmission. All these measures have contributed to the outbreak remaining geographically localised. However, cluster-based management will be more difficult in settings with much more widespread transmission. Finally, the South Korean response was facilitated by structures and processes such as additional legal powers for quarantine, surveillance systems, and digital technologies introduced after the 2015 Middle East respiratory syndrome (MERS) outbreak [79] which may be lacking in other countries. These key aspects may limit the extent to which the testing and contact tracing strategies adopted by South Korea can be translated to other settings.

## Conclusions

South Korea has avoided a large COVID-19 epidemic and has retained the ability to maintain case-based or cluster-based interventions. Individual case-based contact tracing is a relatively minor aspect of their control program, with cluster investigations accounting for two thirds of cases identified. While early adoption of testing and contact tracing are important factors for South Korea’s successful outbreak control, rapid implementation of localised strong social distancing measures in areas with high transmission, border control, and careful monitoring of high-risk contacts are likely to have substantially reduced transmission. At present, it is not possible to estimate the extent to which each measure has contributed to low case counts in South Korea. Some caution is advised in attempting to duplicate their response in other settings with more widespread transmission and higher case numbers.

## Supplementary information

**Additional file 1 **: **Table S1**. A detailed timeline of key events and policy changes throughout the COVID-19 outbreak in South Korea. **Table S2**. Most recent case definitions for suspected cases and patients under investigation for COVID-19 infection (Source: MOHW, last updated June 25th). **Section 2.** Contact tracing of individuals – detailed protocol for contact tracing in South Korea. **Figure S1**. A regional breakdown of the change in epidemiological links of confirmed cases over time. **Table S3**. The prior means and standard deviations explored in our sensitivity analysis of the *R*_*t*_ estimates.

## Data Availability

The data used in this analysis were collated from publicly available KCDC press releases and are made available in a public GitHub repository (https://github.com/mrc-ide/covid19_South_Korea_report) and assigned a permanent DOI on Zenodo (10.5281/zenodo.3978101). In the eventuality that the original links become unstable or cannot be opened by the reader, PDF versions of the KCDC press releases and other governmental webpages referenced in our manuscript have also been uploaded to the repository and can be found using the aforementioned DOI.

## Abbreviations

KCDC: Korea Centers for Disease Control and Prevention
PCR: Polymerase chain reaction
MOHW: Ministry of Health and Welfare
*R*_*t*_: Time-varying reproduction number
RIPHEs: Research Institutes of Public Health and Environment
RCTs: Residential treatment centres
MERS: Middle East respiratory syndrome
