# Supplementary material for: Response to COVID-19 in South Korea and implications for lifting stringent interventions
Source: BMC Med. 2020 Oct 9;18:321. doi: 10.1186/s12916-020-01791-8 (PMC7544529; doi:10.1186/s12916-020-01791-8)
Supplement: Supplementary file 1 — Additional file 1 : Table S1. A detailed timeline of key events and policy changes throughout the COVID-19 outbreak in South Korea. Table S2. Most recent case definitions for suspected cases and patients under investigation for COVID-19 infection (Source: MOHW, last updated June 25th). Section 2. Contact tracing of individuals – detailed protocol for contact tracing in South Korea. Figure S1. A regional breakdown of the change in epidemiological links of confirmed cases over time. Table S3. The prior means and standard deviations explored in our sensitivity analysis of the Rt estimates. [file 12916_2020_1791_MOESM1_ESM.docx]

Additional File 1

Timeline of events and interventions

**Table S1**: Timeline of significant COVID-19 events and interventions for South Korea. Table adapted from [4] up until March 22^nd^ with additions from [7] and the MOHW, MOE and KCDC press releases as referenced. Dates are in 2020, unless stated explicitly.

| **Date** | **Event or Intervention** |
| --- | --- |
| Dec 30, 2019 | Cluster of cases of pneumonia of unknown origin was reported to China National Health Commission |
| Jan 3 | Korean government raised the alert level to Blue (level 1 out of 4-level national crisis management system). Special immigration measures were implemented for those arriving from Wuhan. |
| Jan 12 | Coronavirus was named as 2019-nCoV, and Chinese scientists shared the genetic sequence of the virus internationally. |
| Jan 20 | First confirmed case of Coronavirus reported, a 35-year-old female, Chinese national, residing in Wuhan, Hubei province. Detected with fever upon arrival at the Incheon international airport. Korean government raised the national alert level to Yellow (level 2) |
| Jan 23 | Chinese government locked down Wuhan, the centre of the outbreak. |
| Jan 28 | Korean government raised its infectious disease alert level to Orange (level 3). Special immigration measures were extended to all individuals travelling from mainland China. |
| Jan 30 | WHO declared the coronavirus, global public health emergency |
| Jan 31 | COVID-19 test kits based on the virus' genetic code released by China had been distributed to local government labs across the South Korea. |
| Feb 4 | Korea began banning entry of all foreign nationals who have been to China's Hubei province in the past two weeks |
| Feb 7 | COVID-19 test kits became available in private hospitals. Case definition of a suspected case is expanded. |
| Feb 12 | WHO declared an official name for the new coronavirus - COVID 19 |
| Feb 18 | First case related to Shincheonji cluster was identified |
| Feb 20 | Number of confirmed cases in Korea reached 100, and first death case occurred.  Testing began of the 9,334 members of Shincheonji Daegu group.  Korean government switched to testing anyone with symptoms regardless of travel history or link to an existing case |
| Feb 21 | Korean government declared 'Special Management Region' in Daegu and Cheongdo. Testing of all staff at Cheongdo Daenem hospital was carried out. |
| Feb 23 | Korean government raised its infectious disease alert level to Red (level 4) and delayed the start of the new semester by one week until Mar 9 (schools closed since mid-Feb). School closure Citizens of Daegu asked to refrain from leaving their homes for 2 weeks. |
| Mar 1 | Korean government divided confirmed patients into four groups and only the sickest and elderly were sent to hospitals. The young and asymptomatic went to dormitories, “Life treatment centres”. |
| Mar 2 | Korean government delayed the start of new semester to Mar 23.  7^th^ edition of COVID-19 protocol released with recommendations on self-isolation. |
| Mar 5 | Korean government declared 'Special Management Region', Gyeongsan.  Drive thru sample collection centre established. |
| Mar 9 | Korean government applied special entry procedures for Japan. |
| Mar 10 | A cluster of confirmed cases appeared in a Seoul call centre. |
| Mar 11 | WHO declared COVID-19 a pandemic |
| Mar 17 | Korean government delayed the start of new semester to Apr 6. |
| Mar 19 | Special entry procedures applied for all travellers flying into Korea. |
| Mar 22 | Korean government began implementing stricter rules on social distancing, and began testing all incoming travellers from Europe [18] |
| Mar 27 | Korean government began testing all incoming travellers from the US [18] |
| Apr 1 | Korean government began quarantining all incoming travellers for 14 days and testing them if they develop symptoms. [29] |
| Apr 4 | Korean government extended the period of stricter social distancing until at least the 19^th^ April [30] |
| Apr 9 | Korean government began the new school semester remotely for 3rd year high school and 3rd year middle school students [31]. Pooled testing introduced for testing asymptomatic high-risk groups [32]. |
| Apr 15 | Legislative election held in South Korea |
| Apr 16 | Korean government began the new school semester remotely for 1st and 2nd years in high school, 1st and 2nd years in middle school, and 4th-6th years in elementary school [31]. |
| Apr 19 | Korean government extended the social distancing campaign in a slightly relaxed form until the 5^th^ May [33] |
| Apr 20 | Korean government began the new school semester remotely for 1st-3rd year elementary school students. [31]  A support centre was launched providing information on the development of COVID-19 vaccines, treatments, and prevention/control supplies and equipment |
| Apr 22 | 56 national outdoor facilities re-opened [24] |
| Apr 24 | First meeting of inter-governmental support task force for development of COVID-19 treatment and vaccine held [25] |
| Apr 30 | 14 days since the election and no cases associated with it were found. First day since before February 18th with no new local cases in South Korea [26]. |
| May 6 | Korea starts relaxed “Distancing in Daily Life” social distancing measures [34] |
| May 8 | A cluster of cases confirmed, associated with nightclubs and restaurants visited on 2^nd^ May in Itaewon district of Seoul [27]  Administrative order put in place for nightlife entertainment venues asking them to refrain from opening, and for those that must open, restrictions must still be complied with. [35] |
| May 10 | Korea starts testing all inbound travellers regardless of symptoms [8] |
| May 11 | 8^th^ Edition of the COVID-19 Response Guidelines released. Clinical symptoms in COVID-19 case definition expanded to include: fever, cough, shortness of breath, chills, muscle pain, headache, sore throat, loss of smell or taste, and pneumonia. [35] |
| May 13 | Guidelines on disclosure of travel routes of confirmed cases revised to provide only essential and non-identifiable information to protect privacy. Anonymous testing expanded nationwide. [9] |
| May 18 | Revised management of cases with a recurring positive test following discharge, after investigation shows no infectivity is “re-positive” cases. Testing of discharged cases no longer required prior to return to work.  [28] [11] |
| May 20 | Schools reopen for High School Grade 3. [48] |
| May 25 | Korea began mass testing people who visited public venues and events visited by confirmed COVID-19 cases.  Case definition and reporting put in place for COVID-19 associated Multi-System Inflammatory Syndrome (MIS-C) in children. [10] |
| May 27th | Schools reopen for High School grade 2, Middle School grade 3, Elementary School grades 1 & 2 and Kindergarten. [12] |
| May 28th | Enhanced epidemic control measures implemented for Seoul Metropolitan Region up until the 14^th^ of June (including for residents: postponing social gatherings, avoiding using public venues, and physical distancing >2 metres, and for facilities such as nightlife venues, singing rooms, after-school private academies and PC cafes: subject to gathering ban or allowed limited operation with potential legal action for non-compliance with infection control). [13] |
| Jun 3 | Schools reopen for High School grade 1, Middle School grade 2 and Elementary School grades 3 & 4. [14] |
| Jun 5 | COVID-19 vaccine candidate INOVIO’s INO-4800 approved for domestic clinical trial phase 1 & 2 in South Korea. [15] |
| June 12 | National Health service to cover 50% of the cost of testing new inpatients or residents in high risk facilities in Seoul to help prevent spread. |
| Jun 15 | Enhanced infection control measures in Seoul Metropolitan Region are extended indefinitely, until daily cases are <10. [16] |
| Jun 23 | Direct sale promotion facilities classified as high-risk facilities, requiring them to comply with related regulations ((keep a log of all visitors, wear PPE, disinfect facilities between sales, no performances, singing, or providing meals) [17] |
| Jun 25 | Isolation measures altered: asymptomatic cases released from isolation 10 days after a positive PCR test if they do not develop symptoms or if they have 2 negative PCR tests at least 24h apart 7 days after their positive test. [19] |
| Jul 1 | Entry screening strengthened: screening strengthened for ships, based on whether they originate from high-risk countries, contact levels on-board, risk-level of people leaving the ship and entering Korea. The number of quarantine stations with COVID-19 testing capabilities was increased  Remdesivir began to be supplied to COVID-19 patients [20] |
| Jul 4 | The budget for strengthening research and development investment was confirmed at 191.6 billion won [21] |
| Jul 10 | Korea mandates that all churches comply with infection prevention guidelines (church-related gatherings/events/group meals outside of regular service prohibited, masks should be worn at all times) [22] |
| Jul 13 | Korean government required all foreigners arriving from designated countries subject to strengthened infectious disease prevention and control to be in possession of a negative PCR test that was issued within 48 hours prior to the time of departure. These test results would be submitted upon entry into Korea. [23] |

Detailed protocols for case-based interventions in South Korea

1. *Testing strategy*

Currently all suspected cases and patients under investigation are tested (see most recent definitions in Table S2). Close contacts of confirmed cases are also tested even if they do not exhibit symptoms [66].

Between January 28^th^ and 7^th^ February 7^th^, South Korea increased the number of its screening clinics from 288 to 556 [56]. In addition to traditional screening clinics, selected areas in South Korea also have drive-through and walk-through screening clinics [57, 58]. Both methods offer a quicker turnaround of individuals than traditional methods [57]. Tests require an upper respiratory tract specimen and, if easily provided, a lower respiratory tract specimen [65].

**Table S2:** Most recent case definitions for suspected cases and patients under investigation for COVID-19 infection (Source: MOHW, last updated June 25^th^ [55]).

| **Case type** | **Definition** |
| --- | --- |
| Suspected case | A person displaying clinical symptoms* within 14 days of contact with a confirmed COVID-19 patient. |
| Patient under investigation | 1. A person suspected of COVID-19 according to a physician’s opinion for clinical symptoms* of COVID-19 2. A person with an overseas travel history displaying clinical symptoms* of COVID-19 within 14 days upon entry to Korea 3. A person exhibiting clinical symptoms* of COVID 19 within 14 days with an epidemiological link to a domestic COVID-19 cluster |

*Clinical symptoms of COVID-19 included fever, cough, shortness of breath, chills, muscle pain, headache, sore throat, loss of smell or taste, and pneumonia according to the case definition in the 8^th^ edition of the COVID-19 Response guidelines in South Korea released on May 11^th^ [35].

*2. Contact tracing of individuals*

Contact-tracing in South Korea is a mix of traditional patient interview methods supplemented by access to health data. Contacts of cases are placed in quarantine (their quarantine location depends on their triaged risk group) and an application is used to monitor their movement. Movement restrictions are made under a public health order and release of detailed patient information is allowed under the public health acts introduced in 2015.

According to the Korean CDC [65], the protocol for epidemiological investigations and contact-tracing is divided into four stages:

1. Investigation (collecting information on case’s location history for a specified time period)
2. Exposure risk assessment
3. Contact classification
4. Contact management -- quarantine and symptom monitoring

During the case investigation phase, information collected from traditional patient interviews are then supplemented using mobile phone location data, card transaction logs, and CCTV footage to determine the geographic scope of contact tracing required and to overcome any recall bias. Medical record data, clinical records, and information on the use of medical facilities and pharmacy visits are used to estimate the time-window of infection and time of initial onset of symptoms. If a medical facility is included in the patient’s route, quarantine of the medical facility is conducted [5]. The Epidemiology & Case Management Team (COVID-19 National Emergency Response Center, South Korean Centers for Disease Control) report that these methods are now being used for every confirmed COVID-19 case [63].

The KCDC Coronavirus Disease Response Guidelines specify that family members and close contacts must be identified and quarantined within 24 hours of identifying the confirmed case [66]. For identified contacts, dependent on presence or absence of symptoms and whether they are considered “high risk” they may be immediately isolated in hospitals, asked to quarantine at home, or be transferred to designated quarantine facilities. Regardless of symptoms contacts will then be under “movement restriction” as a public health order [5]. The Ministry of the Interior and Safety have developed a mobile-app [67] that is then used to monitor the movement of those under movement restrictions. Upon case confirmation, mild cases are places in home quarantine, moderate cases in hospital if hospital has capacity, and severe/extremely severe cases in tertiary hospitals [65]. Further information on the epidemiological investigation procedure is available through the Korean Ministry of Health dedicated coronavirus portal [68].

As part of the contact tracing protocol, within 24 hours of identifying a confirmed case, the Municipal COVID-19 Immediate Response Task Force should identify any healthcare or community settings that the case visited during the infectious period (including the day before symptom onset) and conduct an epidemiological investigation. Clusters of cases may be identified this way [66]. On top of contact tracing, South Korea have used targeted mass testing to investigate and manage clusters. In the case of Shincheonji and Guro-gu call centre, the standard case definition of a PUI (Table 2) was adapted to cluster investigation. Rather than explicitly tracing the close contacts of individual cases, local government tested all members of the Shincheonji religious group (42.5% were confirmed as cases) and subsequently monitored them [64]. Similarly, upon being notified of a case that was potentially part of a cluster in a mixed purpose building in central Seoul, every person who had worked resided or visited the building over the three weeks prior to reporting was designated a PUI and tested [63]. 97% of confirmed cases were linked to a call centre of the 11^th^ floor in which the attack rate was calculated as 43%. Close contacts of the confirmed cases discovered by this targeted mass testing were then traced [63]. It is unclear what triggers the use of targeted mass testing over relying exclusively on individual contact tracing in the case of other clusters.

Regional breakdown of case origins and/or epidemiological links


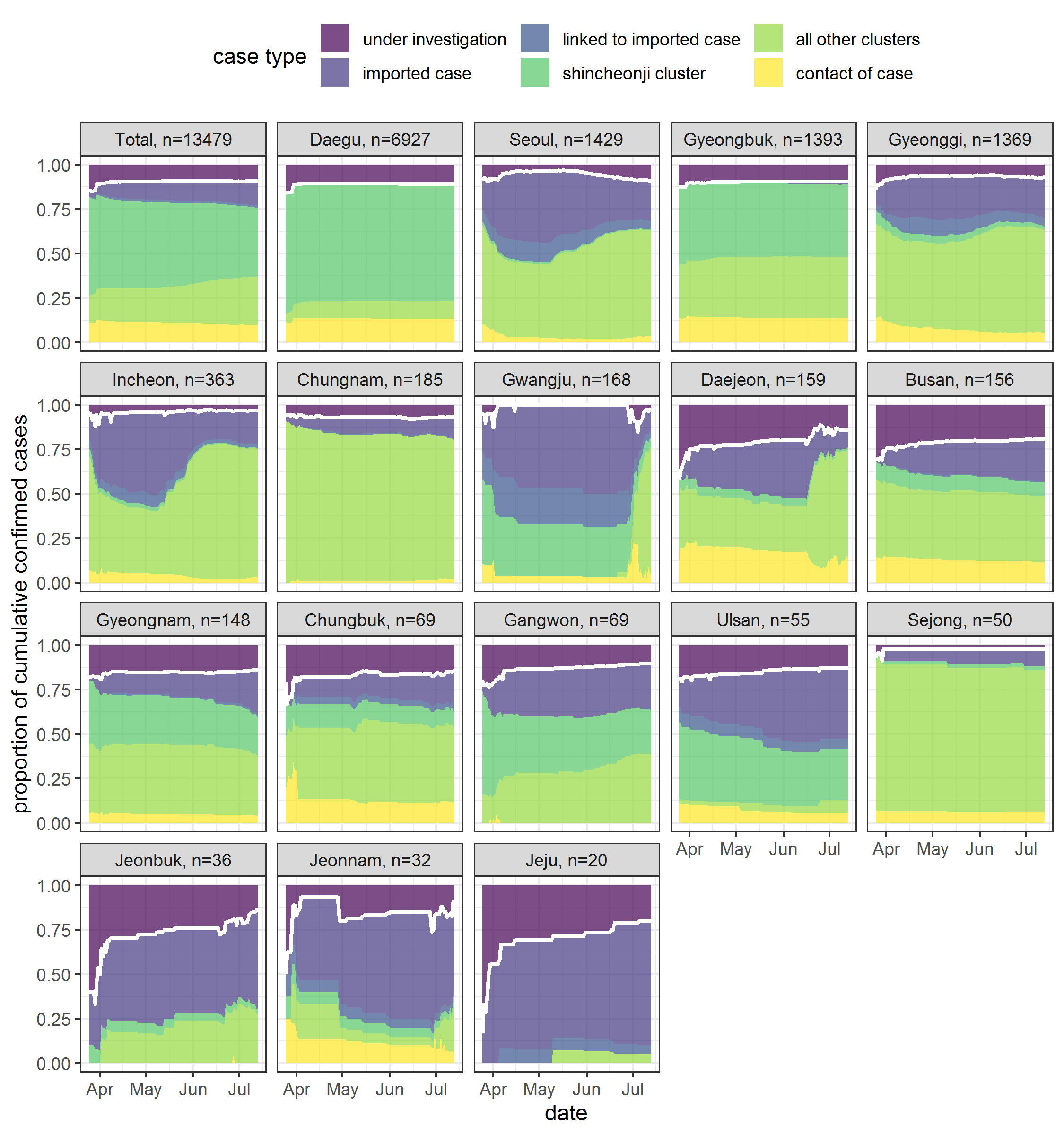


**Figure S1**: Cumulative proportion of confirmed cases by epidemiological link and/or origin from March 25^th^ to July 13^th^ , broken down by region. Regions are in descending order of total number of cases (n). The proportion of cumulative confirmed cases that are linked to existing cases or imported (white line) as opposed to “under investigation” (which includes apparent sporadic cases). Linked cases are broken down into whether they are connected to an imported case, part of the Shincheonji cluster, a smaller cluster or a non-cluster contact of a confirmed case. Source: KCDC press releases as referenced.

*Methods*

**Table S3**: The prior means and standard deviations that were explored to check the sensitivity of our R_t_ estimates.

| **Mean of the prior distribution for *R_t_*** | **Standard deviation of the prior distribution for *R_t_*** |
| --- | --- |
| 5 | 5 |
| 2.6 | 2 |
| 2.6 | 1 |
| 1 | 1 |
| 1 | 0.5 |
| 2.6 | 10 |
| 1 | 10 |
